# Society Meetings

**Published:** 1907-04

**Authors:** 


### SOCIETY MEETINGS

### Buffalo Nurses Association.

[*Trained Nurse and Hospital Review.*]

The annual banquet of the Buffalo Nurses' Association was held the evening of the sixteenth of January, in Miss Vincent's tea rooms, and was one of the most delightful social affairs ever held by that body.

The honor guests were Mrs. Frank Shuler, president of the Western Federation; Dr. Maud J. Frye, an honorary member, and Mrs. Henry Altman, chairman of the educational committee of the City Federation. Much regret was expressed over the absence of Mrs. Annette Sumner Rose, who had been especially invited, and many nurses who had met Mrs. Rose on previous visits to Buffalo hoped to renew the friendship. A telegram was received from Mrs. Rose announcing her inability to be present and was read at the tables, and it was voted that a letter of regret should be sent to her.

The tables were decorated in green and white, tiny pine trees with frost-like effects giving a most pleasing appearance; the favors were medicine glasses filled with green and white confections and tied with green and white ribbons. Place cards with green clover designs and each one with a suitable quotation marked the places.

Between courses the nurses enjoyed dancing Sir Roger de Coverly and apparently a few preferred the dance to the dinner.

Miss Mary Jayne Cole, the president, acted as toastmistress.

Dr. Maud J. Frye responded to the toast "The Patient," as follows:

DR. FRYE'S RESPONSE.

I am to say a few words to you tonight in behalf of the one on whom both nurses and doctors depend for daily bread—the patient.

A very wise physician has said no physician should be allowed to practise medicine till he has had typhoid fever. Acting upon his advice, at the end of my year as a hospital interne I took a two-months' post-graduate course in typhoid. I have never regretted the time I spent. I learned more practical medicine then than in any other two months of my entire student or professional life, and I have once or twice since had occasion to observe the practice of medicine from the patient's standpoint. It is not alone medicine which I have learned during these enforced periods of study. There is no better way to learn nursing.

I would not go so far as to advise a nurse before undertaking her chosen work outside her hospital training school, to deliberately innoculate herself with typhoid, measles, influenza or mumps, merely to make her training complete, but I believe that each and every illness that befalls her, will tend to make her a better nurse. One learns when one's own back is aching why it is sometimes so difficult to adjust the pillows right. The restfulness of absolute quiet will never be appreciated to the full, except by the one who has herself been hurt—from the blows of sound. Then, too, the things you learn about sick-room visitors when you yourself are the visitee! How you would like them all to adopt the motto: "Be Brief." The sensitiveness of the average invalid is something which the well seldom appreciate. Laugh *with* your patient as much as you will, but don't laugh *at* her. Most of all when your own personal and family secrets are of necessity laid bare to stranger eyes, how you will appreciate the nurse who has learned to hold her tongue. How do you know her? A dog who fetches a bone will carry a bone. If your nurse gossips to you of one and another patient and her affairs, will she not also in turn discuss yours?

Your nurses' association serves among other good purposes to interest its members in something outside the sick-room, and I would urge you all to avail yourselves of the opportunities which it affords, and of all other opportunities which offer. for intellectual and social enjoyment and improvement, not only for your own sakes, but that you may be able to interest your patients without being gossips, bright without being malicious. Last and best of all sickness teaches you how much a good nurse comes to be loved by those she cares for. You will never fully understand the mingled feeling of dependence and gratitude and absolute confidence with which it is your privilege sometimes to be regarded, until you have been cared for by a woman worthy of such regard. I have some tender memories treasured up of women who have nursed me and mine in critical illness. Sympathetic without being depressing, cheerful and even merry without being frivolous, brave and unselfish and untiring, their price is above rubies. When you come to take the special course in nursing which I have advised, may you fall into such hands.

Miss Katherine Meagher spoke on "The Helping Hand;" Mrs. J. L. Brodie "The Club-woman," and Mrs. Storck gave a toast which had been prepared by Miss Ten Eyck, but who was unable to be present, "Tell me why when asked a question you will always answer No?"

Miss Mary Swartz responded to the toast "Looking Forward," and Miss Nye spoke on "Looking Backward."

Mrs. Frank Shuler talked on the work of the Western Federation and of the place the nursing profession holds and should hold in civic life. Mrs. Altman told of securing medical inspection in the public schools.

The arrangements for the banquet were made by Mrs. Jennie T. Anderson and Miss Adella Walters.

---

### Medical Society of the County of Erie.

[*Reported by Franklin C. Gram, M. D. Secretary.*]

A SPECIAL MEETING of the Medical Society of the County of Erie was held at the University Club, Buffalo, February 19, 1907, at 4:30 p. m., for the purpose of considering a bill creating a single state board of medical examiners.

After a discussion of the bill, it was moved by Dr. Hubbell, seconded by Dr. Lytle, and

Resolved, that the Medical Society of the County of Erie is in favor of the enactment of Senate Bill No. 154 and Assembly Bill No. 160, which provides a single examining board for the licensing of practitioners of medicine in the state of New York.

The resolution was unanimously adopted.

On motion of Dr. Krauss, the secretary was directed to send a typewritten copy of this resolution, by special delivery, to each member of the Public Health Committee of the senate and assembly.

On motion of Drs. Hubbell and Benedict, Dr. Edward Clark and Dr. William C. Krauss were appointed delegates to represent this society at the hearing in Albany.

Dr. A. A. Hubbell and Dr. F. E. Fronczak were appointed alternates.

On motion of Dr. Bennett, it was voted that the actual expenses of the delegates be paid by the society.

Adjourned.

---

The initial meeting of the Women's Medical Society of the State of New York was held at the Genesee Valley Club, Rochester, the evening of March 11, 1907. There were 48 women physicians in attendance from different sections of the state, 17 of whom were from Buffalo. The proceedings included a banquet at which Dr. Ida C. Bender of Buffalo acted as toastmaster. The chief speaker and guest of honor was Dr. Sarah R. A. Dolley of Rochester, the second woman physician in the state. She was graduated from the Central Medical College of Syracuse and Rochester in 1851.

Among the speakers was Dr. M. Elizabeth Schugens, who spoke on liberty, equality and fraternity. Those attending from Buffalo included Ida C. Bender, who was toastmistress, Mary I. Denton, Elizabeth Dort, Jane North Frear, Maude J. Frye, Edith Hatch, Jeanette P. Himmelsbach, Regina Flood Keyes, Helene Kuhlman, Marion Marsh, Kathrine S. Munhall, Lillian Craig Randall, Electa B. Whipple, Marie S. Wolcott, M. Elizabeth Schugens, Mary H. Sloan, and Amelia Earle Trant.

The preliminary work of organisation was completed by committees and formally adopted.

President, Sarah R. A. Dolley; first vice-president, Electa B. Whipple, Buffalo; second vice-president, Mary H. Cotton, New York; third vice-president, Mary T. Greene, Castile; secretary, Eveline P. Ballentine, Rochester; treasurer, May Allen, Rochester.

---

The Buffalo Academy of Medicine held meetings during the month of March as follows:

*Section on Surgery.*—Tuesday evening, March 5. Program: (a) A working formula for the treatment of intraperitoneal infection, William B. Jones, Attending Surgeon St. Mary's Hospital, Rochester, N. Y.; (b) Typhoid ulceration, John Parmenter.

*Section on Medicine.*—Tuesday evening, March 12. Program: (a) Pleural effusions and a safe and easy means of drainage in purulent cases without resection of rib, Albert J. Colton; (b) Concerning chylo-thorax, Dewitt H. Sherman; (c) Some questions involved in sending a patient away from home, John H. Pryor.

*Section on Obstetrics and Gynecology.*—Tuesday evening, March 19. Program: (a) Postpartum hemorrhage, Ludwig Schroeter; (b) Acute general peritonitis, Francis W. McGuire.

*A Stated Meeting* of the Academy was held Tuesday evening, March 26, at which nominations for officers for ensuing year for president, secretary, treasurer and one trustee was made. The Section on Pathology furnished the program: Formation of gall stones, J. George Adami, of Montreal, Canada.

---

THE NATIONAL ASSOCIATION OF UNITED STATES PENSION EXAMINING SURGEONS will hold it sixth annual meeting at Washington, D. C., during the session of the Seventh Congress of American Physicians and Surgeons, which begins May 7, 1907. At the time this notice is sent to press the precise day of meeting has not been announced, but we conclude that, according to precedent, the sessions will be held Wednesday and Thursday, May 7 and 8.

---

THE AMERICAN CONGRESS OF PHYSICIANS AND SURGEONS will hold its seventh triennial session at Washington, D. C., Tuesday, Wednesday and Thursday, May 7, 8, and 9, 1907. The subject for the second day is, The Comparative Value of Medical and Surgical Treatment of the Immediate and Remote Results of Ulcer of the Stomach, by John H. Musser, Charles G. Stockton, John H. Munro and William J. Mayo. Discussion by Mr. B. G. A. Moynihan, Leeds, and A. Jacobi, New York.

---

THE PHILIPPINE ISLANDS MEDICAL ASSOCIATION held its fourth annual meeting at Manila, February 27, 28, and March 1, 2, 1907. under the presidency of Dr. Paul C. Freer. An elaborate and interesting program was received by the Journal March 25, which courtesy we hereby acknowledge with best thanks.

---

THE AMERICAN MEDICAL ASSOCIATION will hold its sixtieth annual meeting at Atlantic City, Tuesday, Wednesday, and Thursday, and Friday, June 4-7, 1907, under the administration of President William J. Mayo, of Rochester, Minn., and President-elect Joseph D. Bryant, of New York.

---

THE SIXTH INTERNATIONAL DERMATOLOGICAL CONGRESS will be held September 9-14, 1907, at the Academy of Medicine, 17 West 43d Street, New York, under the presidency of Dr. William J. White, of Boston. Dr. Grover W. Wende, of Buffalo, is a member of the organisation committee.

